# Spleen preservation in a caudal pancreatic serous cystadenoma – case report

**Published:** 2015

**Authors:** I Dina, O Ginghina, C Iacobescu, C Vrabie, C Gidea, R Munteanu, R Iosifescu, N Iordache

**Affiliations:** * Surgical Clinic, “Sf. Ioan” Emergency Hospital, Bucharest, Romania; ** Gastroenterology Clinic, “Sf. Ioan” Emergency Hospital, Bucharest, Romania; *** Histology Department, “Sf. Ioan” Emergency Hospital, Bucharest, Romania

**Keywords:** serous cystadenoma, distal pancreatectomy, spleen preservation

## Abstract

Cystic lesions of the pancreas are relatively rare entities but have been increasingly diagnosed in recent years due to advanced imaging techniques. This category encompasses pancreatic pseudocyst as well as a wide range of pancreatic tumors with benign behavior, borderline or primary malignant. Serous cystadenoma of the pancreas represents the most common benign pancreatic tumor, with a very low but well recognized malignant potential. The clinical presentation varies according to its size; small tumors may be asymptomatic and discovered incidentally, while large tumors are more likely symptomatic. We report the case of a female patient presenting with non-specific left abdominal pain, who was diagnosed through a CT scan with a caudal pancreatic tumor. The patient underwent spleen-preserving distal pancreatectomy. The result of the histopathological examination revealed a serous cystadenoma.

## Introduction

Pancreatic cysts are diagnosed with increasing frequency because of the widespread use of cross-sectional imaging. They may be detected in over 2 percent of patients who undergo abdominal imaging with multidetector computed tomography or magnetic resonance imaging for unrelated reasons, and this frequency increases with age [**1**,**2**]. CT scan also represents the most important investigation used to detect a pancreatic mass and to differentiate between a solid lesion and a cystic one. Cystic lesions of the pancreas can be divided into inflammatory fluid collections, non-neoplastic pancreatic cysts, and pancreatic cystic neoplasms (PCNs). Rarely, solid pancreatic tumors may also present as a pancreatic cyst, due to cystic degeneration [**3**]. Benign serous cystadenoma is a pancreatic cystic neoplasm. Distal pancreatectomy is primarily performed for malignant and premalignant diseases of the pancreas. Splenic preservation can be made when treating small neuroendocrine tumors in the body and tail of the pancreas that are likely benign, and premalignant cystic lesions without any objective signs of advanced pathology (e.g. large size, mural nodules, or solid component). There are some obvious haematological advantages when splenic preservation is performed, but some techniques of splenic preservations are also associated with postoperative complications such as splenic infarction [**4**].

## Case report

**Fig. 1:**
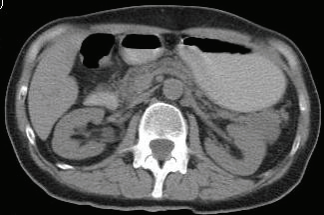
CT aspect of a pancreatic tail tumour

A 62-year-old female presented for continuous diffuse abdominal pain, predominantly located in the left abdominal quadrant, associated with nausea and loss of appetite, without frank weight loss or changes in bowel habit; symptoms started about one month earlier and became progressively worse. The past medical history was significant for mild arterial hypertension controlled with medication and coronary artery disease. Her family history was unremarkable for any medical conditions, and the patient denied alcohol consumption, cigarette smoking or illicit drug use. The clinical examination showed an ill-appearing patient with discrete pallor, normal weighted (BMI = 20 kg/m2). The abdominal examination revealed a slight epigastric sensitivity in the left upper abdominal quadrant, without palpable masses. The laboratory findings (cell blood count, biochemistry, and coagulation profile) were within normal ranges. An abdominal ultrasound was performed but pancreas visualization was not concluding, due to artefaction. An intravenous enhanced abdominal CT scan revealed a well-defined lesion, 35 mm diameter, arising from the tail of the pancreas, hypodense, without contrast enhancement (**Fig. 1**).

**Fig. 2:**
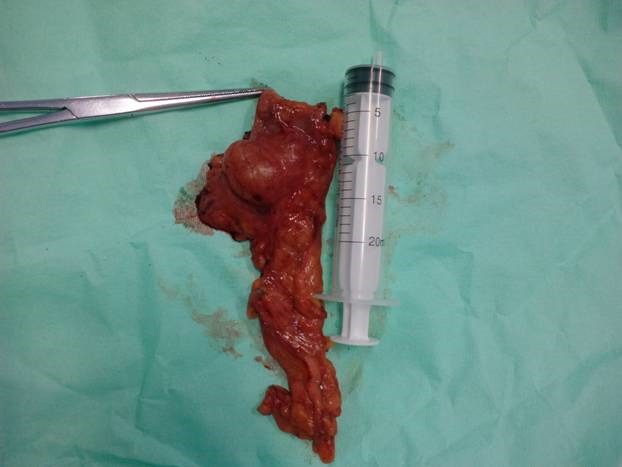
Distal pancreatectomy specimen presenting a cystic lesion – the pathological result established it to be a cystadenoma

Serum tumor markers, CA19-9 and CEA, were normal. The patient was referred to surgery, where a distal pancreatectomy with spleen preservation was performed (**Fig. 2**), since the abdominal exploration showed no local invasion or distant metastases.

**Fig. 3:**
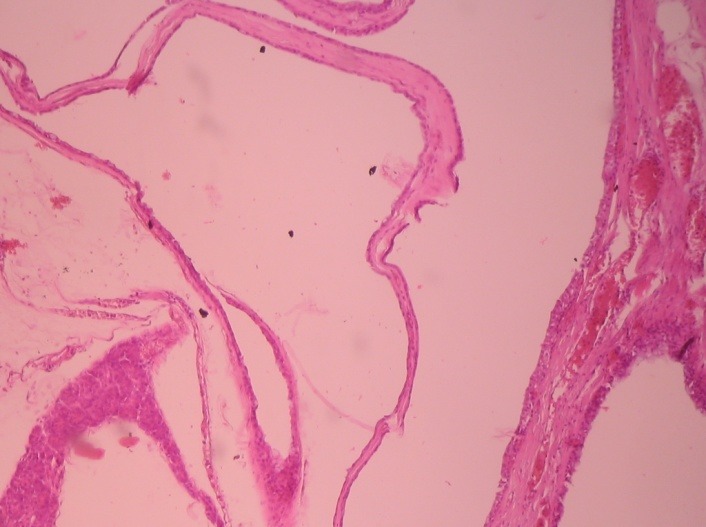
Wall cyst of the pancreas with flat epithelium, HEX10

**Fig. 4:**
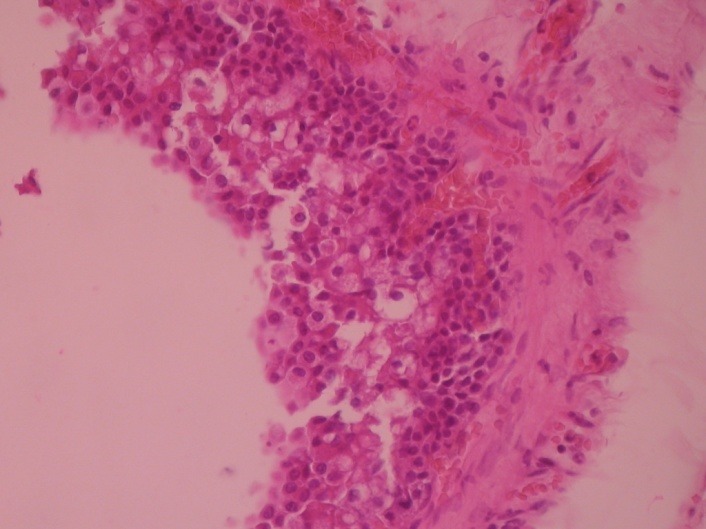
Wall cyst of the pancreas with epithelium hyperplasia, mixted with xantomatous cells, HEX20

After closing the main pancreatic duct with separate sutures, a running suture was used to close the residual transected pancreatic parenchyma. In order to check for pancreatic leakage, the amylase level in the abdominal drain was measured starting with the second postoperative day, and it showed a decreased value. The patient had an uneventful postoperative course, with a marked improvement of her general state at follow-up visits (3 and 6 month after the procedure). Moreover, a Doppler ultrasound of the spleen was performed to confirm a proper vascularization during follow-up. The histopathologic finding was consistent with a benign serous cystadenoma, with frequent dilated pancreatic ducts, some of them with cystic appearance, lined by cuboidal epithelial cells (**Fig. 3**,**4**).

## Discussions

Accurate cyst classification is important since non-neoplastic cysts require treatment only if symptomatic, whereas some of the pancreatic cystic neoplasms have significant malignant potential and should be resected. Inflammatory fluid collections are not true epithelial cysts and typically represent local complications of acute pancreatitis. Non-neoplastic pancreatic cysts (NNPCs) include a variety of very rare cysts that are often asymptomatic and do not require resection. These include true cysts, retention cysts, mucinous non-neoplastic cysts, and lymphoepithelial cysts. They are typically diagnosed after the surgical resection of a lesion that was thought to be a pancreatic cystic neoplasm (PCN) preoperatively. Identifying pancreatic cystic neoplasms (PCNs) is important, since some have malignant potential. PCNs are categorized by using the WHO histological classification: serous cystic tumors, mucinous cystic neoplasms (MCNs), intraductal papillary mucinous neoplasms (IPMNs) and solid pseudopapillary neoplasms (SPNs). Each of the subtypes has benign and malignant forms [**3**]. Serous cystadenomas are the most common type of cystic neoplasm of the pancreas and have a natural history and malignant potential different from that of other cystic neoplasms. Although characteristic findings on the imaging may be supportive, the definitive diagnosis of these lesions cannot often be made by imaging alone. The endoscopic ultrasound with fine needle aspiration and cyst aspiration could facilitate the diagnosis, and after a definitive diagnosis, patients with lesions that are small and asymptomatic may be followed with serial imaging. The management dilemma arises if definitive diagnosis cannot be made or if the patient is symptomatic. At the time, most of the literature evidences support surgical resection for large and/ or symptomatic cystadenomas [**5**-**7**]. The proper resection for caudal pancreatic serous cystadenoma is distal pancreatectomy. Spleen-preserving distal pancreatectomy is indicated for lesions confined to the pancreas [**8**]. Preservation of the spleen in distal pancreatectomy has recently attracted considerable attention. Splenic preservation is a predictive factor for postoperative morbidity after distal pancreatectomy [**9**]. Spleen-preserving distal pancreatectomy (SPDP) is associated with lower postoperative morbidity than distal pancreatectomy with splenectomy [**10**]. In SPDP, a very slight elevation of the platelet count in serum may help to prevent the infarction of the lungs and brain compared to distal pancreatectomy with splenectomy [**4**]. Two major spleen-preserving procedures reported are the Warshaw procedure that conserves the spleen by blood flow from the short gastric vessels and the Kimura procedure that preserves the spleen with splenic vessels [**8**]. Warshaw procedure is technically easier to perform than spleen preserving distal pancreatectomy, but has a higher incidence of subsequent splenectomies. Surgeons should be able to perform both procedures and choose the technique according to the patient [**11**]. Another advantage of SPDP performed according to Kimura procedure is that the blood supply to the proximal stomach is conserved in patients with SPDP who undergo distal gastrectomy with resection of the left gastric artery. On the other side, there is a risk for splenic infarction when it is preserved and most of the complications are associated with the Warsaw operation [**10**].

## Conclusions

Serous cystadenoma is a pancreatic cystic neoplasm, which can have either a benign or a malignant form. When definitive diagnosis cannot be made or if the patient is symptomatic, or the cyst is larger than 4cm, evidences support surgical resection. Benign lesions, as well as low-grade malignancy of the body and tail of the pancreas, may be indications for this spleen preserving distal pancreatectomy.
